# The SIESTA Trial: A Randomized Study Investigating the Efficacy, Safety, and Tolerability of Acupressure versus Sham Therapy for Improving Sleep Quality in Patients with End-Stage Kidney Disease on Hemodialysis

**DOI:** 10.1155/2017/7570352

**Published:** 2017-02-21

**Authors:** Kunyu Shen, Yeoungjee Cho, Elaine M. Pascoe, Carmel M. Hawley, Veronica Oliver, Kathryn M. Hughes, Richard Baer, Jeremy Frazier, Elizabeth Jarvis, Ken-Soon Tan, Xusheng Liu, Glenda Gobe, David W. Johnson

**Affiliations:** ^1^Department of Nephrology, Princess Alexandra Hospital, Brisbane, QLD, Australia; ^2^Australasian Kidney Trials Network, The University of Queensland, Brisbane, QLD, Australia; ^3^Department of Nephrology, Guangdong Provincial Hospital of Chinese Medicine, Guangzhou, China; ^4^Second School of Clinical Medicine, Guangzhou University of Chinese Medicine, Guangzhou, China; ^5^Department of Nephrology, Logan Hospital, Logan, QLD, Australia; ^6^School of Medicine, Griffith University, Brisbane, QLD, Australia; ^7^School of Medicine, The University of Queensland, Brisbane, QLD, Australia; ^8^Centre for Kidney Disease Research, School of Medicine, The University of Queensland, Translational Research Institute, Woolloongabba, Brisbane, QLD, Australia

## Abstract

*Objectives*. To compare the effectiveness of real acupressure versus sham acupressure therapy in improving sleep quality in patients receiving hemodialysis (HD) or hemodiafiltration (HDF).* Methods*. A multicenter, single-blind, randomized controlled trial was conducted in two Australian dialysis units located in Princess Alexandra Hospital and Logan Hospital, respectively. Forty-two subjects with self-reported poor sleep quality were randomly assigned to real (*n* = 21) or sham (*n* = 21) acupressure therapy delivered thrice weekly for four consecutive weeks during routine dialysis sessions. The primary outcome was the Pittsburgh Sleep Quality Index (PSQI) score measured at week four adjusted for baseline PSQI measurements. Secondary outcomes were quality of life (QOL) (SF-8), adverse events, and patient acceptability (treatment acceptability questionnaire, TAQ).* Results*. The two groups were comparable on global PSQI scores (difference 0.19, 95% confidence interval [CI] −1.32 to 1.70) and on the subscale scores. Similar results were observed for QOL both in the mental (difference −3.88, 95% CI −8.63 to 0.87) and the physical scores (difference 2.45, 95% CI −1.69 to 6.58). There were no treatment-related adverse events and acupressure was perceived favorably by participants.* Conclusion*. Acupressure is a safe, well-tolerated, and highly acceptable therapy in adult hemodialysis patients in a Western healthcare setting with uncertain implications for therapeutic efficacy.

## 1. Introduction

Self-reported poor sleep quality is exceedingly common in patients with chronic kidney disease, particularly in those with end-stage kidney disease requiring maintenance hemodialysis where reported prevalence rates are as high as 80% [1–8]. The high frequency of poor sleep quality in hemodialysis patients has been attributed to a lack of nocturnal endogenous melatonin surge [9], increased core body temperature from exposure to warm dialysate [10], and possibly cerebral edema in the context of dialysis disequilibrium syndrome [11]. Other risk factors for poor sleep quality reported by the Dialysis Outcomes and Practice Pattern study (DOPPS) include the use of certain types of medications (e.g., antihistamine, antidepressant, anti-inflammatory, antiasthmatic, narcotic, gastrointestinal, or hypnotics), female gender, higher body mass index (BMI), presence of body pain or pruritus, comorbidities (e.g., depression, lung disease, and peripheral arterial disease), high serum phosphorus levels, and unfavorable lifestyle behaviors (e.g., lack of exercise and smoking) [12]. Poor sleep quality is in turn associated with heightened risk of hypertension [13], chronic kidney disease progression [14, 15], impaired glucose tolerance [16], diabetes mellitus [17], cardiovascular events [18], depression [19], increased healthcare utilization [20], impaired quality of life (QOL), and mortality [12].

Current therapeutic interventions purported to improve sleep quality in dialysis patients include pharmacotherapy with hypnotic agents [21] and melatonin [22], cognitive-behavioral therapy [23] (e.g., sleep hygiene [24], relaxation [25], mindfulness meditation [26], and stationary cycling [27]), nocturnal hemodialysis conversion [28], and cooled (35°C) dialysate use [29]. These interventions have been limited by poor quality evidence base, a lack of demonstration of consistent or sustainable benefit, frequent side effects (particularly for pharmacologic agents in the setting of impaired renal function exposed to polypharmacy), and a high cost burden.

Alternatively, acupressure applied at specific meridians or acupoints has been employed in Traditional Chinese Medicine to improve sleep quality by restoring the yin and yang harmony and balancing life energy (qi) [30]. Several studies have demonstrated its safety and efficacy in various patient populations, including the elderly [31–34], postmenopausal women [35], and cancer patients [36]. Acupressure is a noninvasive therapy, which is likely to be associated with a low risk of side effect profile, when compared to therapies such as hypnotics.

To date, there have only been three randomized controlled trials examining the effect of acupressure therapy in HD patients with demonstrated benefit in improving quality of sleep. First, Shariati and colleagues reported a significant reduction in PSQI scores in hemodialysis patients who received thrice weekly acupressure treatment compared to those who received routine therapy in their 4-week study involving 48 end-stage kidney disease patients (*p* < 0.001) [37]. Although the investigators described this study as a double-blind randomized controlled trial, the control group received no treatment beyond their routine care; therefore the participants would have known their treatment allocation status, which raises concern for “placebo” effect. Similarly, a significant reduction in the PSQI scores was observed in a multicenter randomized controlled trial from Taiwan when acupressure therapy was superior compared to routine care (*p* < 0.01), with comparable results between real and sham acupressure treatment groups [38]. Although this study randomized 105 participants, there was no description of number of patients allocated to each treatment group nor the reasons for those who did not complete the study (*n* = 7) or safety data. Neither of these studies adequately described the methods of randomization nor allocation concealment, which places them at a high risk of bias from suboptimal methodological quality. The most recent randomized controlled trial, conducted by Arab and colleagues in 108 prevalent hemodialysis patients from 3 Iranian hemodialysis units, reported significantly lower total PSQI scores at 4 weeks in patients treated with acupressure compared with sham acupressure or no treatment (*p* < 0.001) [39]. Nonetheless, the study recruited patients with PSQI scores greater than 5, which may have limited generalization of their findings. In fact, all the above-mentioned studies excluded patients with an entry PSQI score less than 5, which may have limited the generalizability of their findings to patients with self-reported poor sleep quality who may have recorded lower PSQI scores. Moreover, all studies were conducted in Asian countries, such that the generalizability of their findings and acceptability of such treatment modality to Western countries remain unclear.

## 2. Objects

The aims of this multicenter, single-blind, parallel design randomized controlled trial were to (1) investigate the effect of acupressure on the sleep quality of prevalent hemodialysis patients and (2) establish the feasibility and safety of acupressure treatment in a Western healthcare setting.

## 3. Methods

### 3.1. Study Oversight

The SIESTA (Study Investigating the Efficacy, Safety, and Tolerability of Acupressure versus sham therapy for improving sleep quality in patients with end-stage kidney disease on hemodialysis) study was an investigator-initiated, multicenter, prospective, 1 : 1 randomized, single-blind, sham-controlled, parallel design trial. Participants were recruited from the dialysis units at Princess Alexandra Hospital and Logan Hospital, Australia. This study was registered with the Australian New Zealand Clinical Trials Registry (ACTRN 12615000989549) and the study protocol was approved by ethics committees at both participating centers. All patients provided written participant information and consent form (PICF) before trial participation and the trial was conducted in accordance with the principles of the International Conference on Harmonization Good Clinical Practice Guidelines [40].

### 3.2. Participants

The study included adult patients (age >18 years) with end-stage kidney disease and self-reported poor sleep quality who were receiving thrice weekly hemodialysis or hemodiafiltration for at least 3 months prior to study enrolment and had no plans to change their renal replacement modality within the study period. All patients had to have sufficient knowledge of English to be able to complete questionnaires independently. English language proficiency of participants who were nonnative English speakers was assessed by clinical staff. Exclusion criteria included those with a history of amputation (precluding ability to apply acupressure treatment on limbs), serious skin diseases (e.g., dermatitis and burn) near the location of acupoints, and previous knowledge of the Traditional Chinese Medicine to minimize the risk of participants being able to distinguish the real acupoints from the sham or the nonspecific acupoint. Recruitment occurred between 23rd September 2015 and 2nd December 2015.

### 3.3. Study Treatment

After enrolment, participants were asked to keep a sleep diary for one week to collect information on sleep duration, quality, reasons for sleep disturbance, and habits that may influence sleep quality (e.g., caffeine consumption; Supplementary Appendix 1 in Supplementary Material available online at https://doi.org/10.1155/2017/7570352). At the baseline visit prior to commencement of study treatment, patients were asked to independently complete the PSQI (Supplementary Appendix 2) [41] and short form-8 (SF-8) Health Survey (Supplementary Appendix 3) [42]. Patients were randomized 1 : 1 to receive either acupressure therapy (intervention) or sham acupressure therapy (control) delivered thrice weekly for four consecutive weeks during their routine dialysis sessions (morning, afternoon, or evening). Participants in the intervention group received acupressure therapy on Shen Men (HT7) and Yongquan (K11); participants in the control group received sham acupressure therapy on a nonspecific acupoint, Zhongquan (EX-EU3), and a sham acupoint (S) (Supplementary Appendix 4). All selected acupoints were stimulated bilaterally. One accredited Traditional Chinese Medicine health practitioner applied acupressure treatment in all participants of the study.

To ensure adequate concealment of allocation, a computer-generated randomization schedule was kept in sequentially numbered, sealed opaque envelopes. Study participants, care providers working in the dialysis units, an outcome assessor, and study statistician were blinded to the participant's allocation to minimize the potential for performance bias. Only the Traditional Chinese Medicine health practitioner delivering acupressure was unblinded and was prohibited from revealing the treatment allocation status.

The therapy was applied at each acupoint for 3 minutes and the applied intensity was adjusted according to the patient's level of tolerance. At the conclusion of the 4th study week, all participants were asked to complete a sleep diary, SF-8, PSQI, and treatment acceptability questionnaire (TAQ) (Supplementary Appendix 5) [43]. During the study period, participants were instructed to not make any adjustments to preexisting sleeping aides (e.g., doses of hypnotics, yoga, and tai chi).

### 3.4. Study Outcomes

The primary outcome was PSQI global and subscale scores at four weeks. Prespecified secondary outcomes were QOL measured using the SF-8 at four weeks, patient acceptability, and adverse events. The SF-8 Health Survey includes a total of eight questionnaire items and incorporates two dimensions: (i) a mental component summary (MCS) which reflects mental health, role emotional, and social function and (ii) a physical component summary (PCS) which reflects physical function and health (license number: QM 034204) [42].

### 3.5. Statistical Analysis

Baseline characteristics are expressed as frequencies (percentages) for categorical variables, mean ± SD for continuous normally distributed variables, and median (interquartile range) for continuous nonnormally distributed variables. Analysis of covariance (ANCOVA) was used to examine the differences in treatment effects between the intervention and control groups adjusted for baseline measurements. Week four PSQI global scores, MCS and PCS of SF-8, and all the subscales of the PSQI were analyzed using ANCOVA. The normality assumption was assessed by examining residuals from the ANCOVA models. The assumption of homogeneity of within-group regression coefficients was assessed by testing the significance of the treatment group by baseline measurement interaction. Sensitivity analyses were performed by including as covariates in the models two baseline characteristics on which the two groups were statistically significantly different (duration of dialysis and hours per dialysis session). Statistical analyses were performed using Stata/MP 14 (College Station, TX, USA) statistical software. *p* values of <0.05 were considered to represent a statistically significant result.

### 3.6. Sample Size

The SIESTA study was a pilot study designed to examine the efficacy and acceptability of acupressure as a therapy in hemodialysis and hemodiafiltration patients in Australia. Therefore, no formal sample size calculation was conducted. The study aimed to recruit at least 40 patients, a feasible number considering patient numbers at the dialysis units and the workload of the practitioner who would deliver the therapy.

## 4. Results

### 4.1. Patients

**Figure 1:**
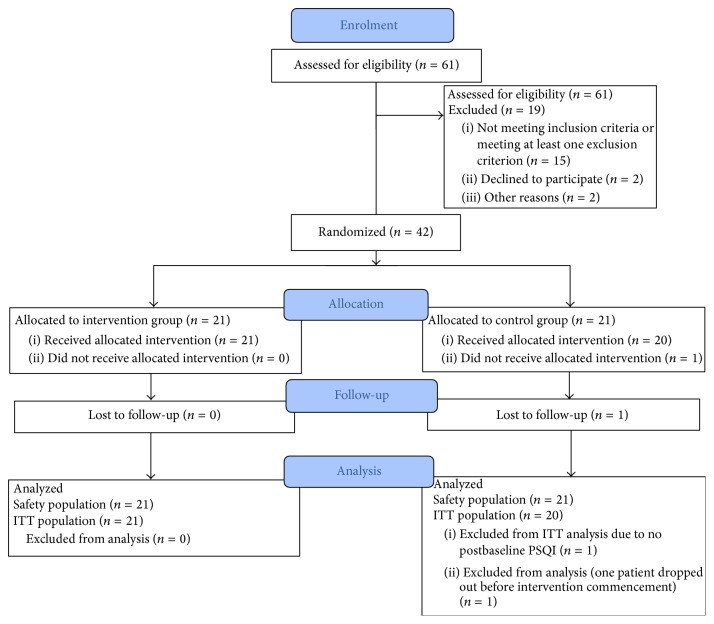
Flow chart of patient progression through the trial. ITT: intention to treat analysis.

Forty-two patients were randomly assigned to receive either real acupressure therapy (*n* = 21) or sham acupressure therapy (control: *n* = 21; Figure 1). One participant declined to participate after randomization but prior to receiving any treatment. Overall, the groups were comparable on baseline characteristics except for significantly longer dialysis duration (46 months versus 21.5 months, *p* = 0.04) and duration of each dialysis session (5.2 ± 0.5 versus 4.7 ± 0.6 hours, *p* = 0.01) in the intervention group (Table 1). At baseline, the intervention group patients reported a significantly higher frequency of experiencing days with no chance of daytime somnolence compared to the control group (median 4.5 versus 0.5, *p* = 0.03; Supplementary Table S1). Other parameters reported in the sleep diary were comparable between the two groups (i.e., sleep duration, ability to fall asleep, mood, exercise, frequency of daytime naps, and alcohol or caffeine consumption).

### 4.2. Pittsburg Sleep Quality Index

**Figure 2:**
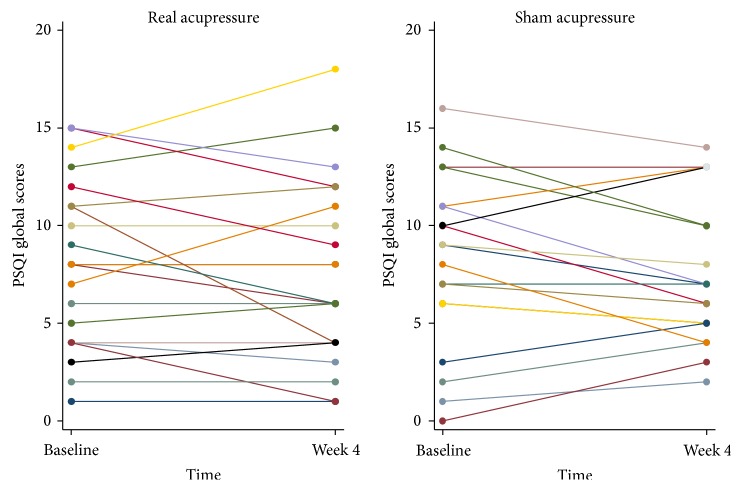
Pittsburgh Sleep Quality Index global scores at baseline and at week 4 in real acupressure (*n* = 21) and sham acupressure (*n* = 20) groups.

In general, both groups demonstrated a reduction in global PSQI scores from baseline to week four (Figure 2, Table 2). However, these differences were not statistically significant (intervention: difference 0.52, 95% CI −1.7 to 0.61, *p* = 0.35; control: difference 0.74, 95% CI −1.91 to 0.43, *p* = 0.20). Moreover, differences between the intervention and control groups on PSQI global scores (difference 0.19, 95% CI −1.32 to 1.70, *p* = 0.8) and the PSQI subscales were small and none of the observed results were statistically significant after adjustment for baseline values (Table 2). Results were similar after additional adjustment for baseline characteristics on which the two groups differed (i.e., months on dialysis and duration of treatment at each session) (Supplementary Table S2).

### 4.3. Quality of Life

The intervention and control groups were similar on the two QOL measures at four weeks after adjustment for baseline values (mental component summary [MCS]: difference −3.88, 95% CI −8.63 to 0.87, *p* = 0.11; physical component summary [PCS]: difference 2.45, 95% CI −1.69 to 6.58, *p* = 0.24; Table 2) and after further adjustment for baseline months on dialysis and duration of treatment at each dialysis session (Supplementary Table S2).

### 4.4. Treatment Acceptability

Acupressure therapy was rated as highly acceptable by both intervention and control group patients (median score 7 versus 6, *p* = 0.41; Table 3). There was no significant difference in perceived efficacy (*p* = 0.9), side effects (*p* = 0.43), or trust in the competency of the Traditional Chinese Medicine health practitioner delivering therapy (*p* = 0.23).

### 4.5. Adverse Events

There were six adverse events (6 participants) recorded during the study (Table 4), all of which were rated as serious adverse events (SAEs) as they led to hospitalization. Two SAEs occurred in the intervention group (fluid overload and ocular hemorrhage) and the remaining four SAEs occurred in the control group (necrotizing fasciitis, physical trauma, chest muscle pain, and arteriovenous graft failure). No adverse event was considered by investigators to be causally related to the study intervention. No local skin reaction (e.g., bruise) from repeated acupressure was reported during the study.

## 5. Discussion

This study demonstrated that, when compared with sham therapy, four weeks of acupressure did not significantly improve sleep quality or quality of life in patients on maintenance hemodialysis or hemodiafiltration. Nevertheless, acupressure was found to be a safe, well-tolerated, and acceptable form of treatment by study participants.

These findings are in keeping with the outcome of a randomized controlled trial conducted in 105 adult hemodialysis patients from four Taiwanese centers where acupressure did not significantly decrease PSQI scores compared with sham therapy after 4 weeks [38]. A lack of benefit in improving sleep quality measured using PSQI from acupressure therapy compared to sham therapy was again observed in the present study. These results contrast with findings from Arab and colleagues, who conducted a single-blind randomized controlled trial in 108 prevalent hemodialysis patients in Iran [39]. Participants were randomized to three groups (acupressure, sham acupressure, and a control group who received routine care only). Total PSQI scores at the end of 4 weeks were significantly lower in the acupressure group compared to the other two groups. Unfortunately, the analysis was conducted on a per-protocol basis; the dropout rate was moderate (14%), and the statistical methods employed did not adjust for baseline differences in PSQI global scores [44, 45]. Other previous studies have reported beneficial effects of acupressure on insomnia in patients without chronic kidney disease, including the elderly [31, 33], postmenopausal women [35], and cancer patients [36]. The apparent disparity in findings compared with the present study may relate to the limitations of the previous studies, including suboptimal methodologic quality, for example, lacking of placebo control [35, 36], selective outcome reporting [31], and inclusion of predominantly Asian patient populations [31, 33, 35, 36].

From a Traditional Chinese Medicine perspective, insomnia may arise in end-stage kidney disease patients because of a dynamic disequilibrium between the kidney (yin or water) and heart (yang or fire), such that the exhaustion of the kidney yin leads to a failure of the kidney water to restrict the heart fire [46]. Acupressure applied to the HT7 (heart meridian) and K11 (kidney meridian) is thought to restore yin and yang harmony (important for normal sleep-wake rhythm) by adjusting the disturbance of the kidney and heart. However, the mechanism of insomnia is likely to be different between individuals from a Traditional Chinese Medicine perspective. For instance, besides the yin-yang disequilibrium which is the holistic mechanism of insomnia, there are other conditions known to promote insomnia, such as the deficiency of the “Qi and blood” of the heart and spleen, and the internal disturbance of pyrophlegm [47]. For each patient diagnosed with specific conditions, the optimal acupoints, intensity, and frequency of stimulation are different. However, in order to standardize the delivery of treatment, all patients received treatments at the same acupoints based on their treatment allocation status in the present study. Therefore, this “one size fits all” approach could have potentially blunted the real general effects of the individualized acupressure therapy, which is utilized in a real-world setting.

From a Western medicine perspective, the mechanism underpinning any beneficial effects of acupressure is uncertain but may relate to an effect of massage on relaxation [48]. Alternatively, acupressure applied to acupoints may promote relaxation through the stimulation of neurotransmitters, such as serotonin [49].

The strengths of this study include concealed random allocation of treatments, involvement of two centers, use of a sham therapy control, examination of safety and treatment acceptability, blinding of outcome assessors, broad inclusion criteria (i.e., patients with self-reported poor sleep quality independent of PSQI scores were eligible), and robust statistical adjustment for baseline values. These strengths should be balanced against the trial's limitations, including a lack of blinding of the therapist delivering treatment, which could have introduced the risk of performance bias. However, the therapist was prohibited from disclosing the treatment allocation status, and data from questionnaires were independently assessed by a blinded outcome assessor (YC), to further reduce the risk of bias. As this was a pilot study, the sample size was small which could have introduced the risk of type 2 statistical error. However, the observed effect size between the two treatment groups was small (mean difference 0.19; Table 2) and unlikely to be considered a clinically meaningful improvement. Nonetheless, therapy was delivered thrice weekly during dialysis sessions for four weeks, and it remains unknown whether an increase in therapy frequency (e.g., daily) or duration (e.g., eight weeks) or nocturnal treatment would result in clinically significant benefit. Indeed, there is limited, low quality evidence suggesting that the use of overnight wrist compression with acupressure devices has beneficial effects on sleep quality in patients without kidney disease [50]. The acupressure points chosen for the intervention group may also not have been the most appropriate point to help improve insomnia at an individual level, and sham acupressure points could have had a positive impact on insomnia. In addition, decreasing global PSQI scores in both intervention and sham groups were observed, which is consistent with the placebo effect from receiving “therapy.” Finally, individualized therapy is a major characteristic of Traditional Chinese Medicine. However, it is difficult to incorporate this level of individualization of therapy into practice in a randomized controlled trial which evaluates the response of a group of individuals to a “one size fits all” therapeutic approach.

## 6. Conclusion

In conclusion, the present pilot study identified that acupressure is a safe, well-tolerated, and highly acceptable therapy in adult hemodialysis patients in a Western healthcare setting but did not result in any clinically meaningful improvement in sleep quality or quality of life compared with sham therapy.

## Supplementary Material

The supplementary material shows all the questionnaires that were employed in the SIESTA study. Supplementary appendix 1 shows the sleep diary designed to reflect sleep patterns of patients on haemodialysis, including elements that may disturb their nocturnal sleep (i.e. pain and caffeine intake) in Supplementary Table S1. The Pittsburg Sleep Quality Index (PSQI) instrument is found in supplementary appendix 2; and the Short form-8 (SF-8) Health Survey Scoring Demonstration in supplementary appendix 3. Indications of acupoints stimulated in the study are summarized in a figures clarifying the locations, in supplementary appendix 4, and the Treatment Acceptability Questionnaire (TAQ) is found in supplementary appendix 5. The results of the treatment group differences in the main and secondary outcomes when additionally adjusted for another two confounding factors, duration of dialysis and hours per dialysis session, are in Supplementary Table S2.

## Figures and Tables

**Table 1:** Baseline characteristics of treatment group.

Characteristics	Intervention (*n* = 21)	Control (*n* = 20)
Age (years)	58.6 ± 11.9	51.6 ± 17.9
Female	6 (28.6%)	9 (45%)
Ethnicity		
Caucasian	11 (52%)	13 (65%)
Aboriginal and Torres	3 (14%)	1 (5%)
Asian	1 (5%)	2 (10%)
Maori and Pacific Islander	6 (29%)	4 (20%)
Marital status		
Married or living with a partner	10 (48%)	11 (55%)
Single	11 (52%)	9 (45%)
Education status		
Less than high school	5 (24%)	3 (15%)
High school finished	16 (76%)	17 (85%)
Cause of end-stage kidney stage		
Diabetes	8 (38%)	7 (35%)
Glomerulonephritis	2 (10%)	5 (25%)
Hypertension	4 (19%)	1 (5%)
Reflux nephropathy	2 (10%)	1 (5%)
Polycystic kidneys	1 (5%)	1 (5%)
Other	4 (19%)	5 (25%)
Comorbidity		
Coronary artery disease	10 (48%)	4 (20%)
Diabetes	8 (38%)	12 (60%)
Hypertension	13 (62%)	11 (55%)
Congestive heart failure	2 (10%)	2 (10%)
Peripheral vascular disease	6 (29%)	3 (15%)
Cerebrovascular disease	3 (14%)	3 (15%)
Chronic lung disease	12 (57%)	10 (50%)
PLMD/RLS	9 (43%)	6 (30%)
Insomnia	2 (10%)	1 (5%)
Mood disorder	11 (52%)	6 (30%)
Gastroduodenal ulcer or reflux	7 (33%)	10 (50%)
Treatment type		
HD	2 (10%)	2 (10%)
HDF	19 (90%)	18 (90%)
Dialysis duration (months)	46 [28, 116]	21.5 [13, 56.5]
Time of day to have dialysis		
Morning	15 (71%)	13 (65%)
Afternoon	6 (29%)	6 (30%)
Evening	0 (0%)	1 (5%)
Hours per dialysis session	5.2 ± 0.5	4.7 ± 0.6
Medications		
Antidepressants	5 (24%)	3 (15%)
Hypnotics	3 (14%)	1 (5%)
Bronchodilators	4 (19%)	1 (5%)
Antihistamines	2 (10%)	1 (5%)
Proton pump inhibitors	7 (33%)	7 (35%)
Nonpharmaceutical interventions to aid sleep		
Exercise	0 (0%)	4 (20%)
Massage	1 (5%)	0 (0%)
CPAP machine	2 (10%)	0 (0%)
None	18 (86%)	16 (80%)
BMI (kg/m^2^)	32.9 [26.5, 35.3]	29.65 [21.2, 34]
Pre-SBP (mmHg)	148.6 ± 27.5	149.7 ± 23.0
Post-DBP (mmHg)	79 ± 16.2	73.6 ± 22.3
Hemoglobin (g/L)	116.3 ± 17.1	112.8 ± 10.5
Phosphate (mmol/L)	2.0 ± 0.6	2.0 ± 0.6
Urea reduction ratio^a^	0.8 ± 0.1	0.8 ± 0.1
KT/V^b^	1.4 [1.2, 1.5]	1.3 [1.2, 1.7]
PSQI global score	8.1 ± 4.3	8.3 ± 4.4
SF-8		
Physical component summary (PCS)	43.3 ± 11.5	45.0 ± 10.6
Mental component summary (MCS)	45.9 ± 13.6	48.5 ± 10.6

BMI, body mass index; DBP, diastolic blood pressure; HD, hemodialysis; HDF, hemodiafiltration; PSQI, Pittsburgh Sleep Quality Index; SBP, systolic blood pressure; SF-8, Short Form-8 Health Survey; PMLD, periodic movement disorder; RLS, restless legs syndrome; CPAP, continuous positive airway pressure.

*Note*. Values for categorical variables are given as number (percentage); values for continuous variables are given as mean ± SD if normally distributed or if nonnormally as median [interquartile range]. ^a^One missing value in the control group at baseline. ^b^One missing value in the intervention group at baseline.

**Table 2:** Primary (PSQI) and secondary (QOL) outcomes at four weeks of treatment group (adjusted for baseline values).

Outcome	Intervention (*N* = 21)	Control (*N* = 19)	Difference (intervention − control) [95% CI]	*p* value
*PSQI global scores*	7.62	7.42	0.19 [−1.32 to 1.70]	0.80
^*∗*^ *PSQI subscales*				
Sleep duration	1.06	1.08	−0.01 [−0.40 to 0.38]	0.95
Habitual sleep efficiency	1.12	1.23	−0.11 [−0.72 to 0.50]	0.73
Subjective sleep quality	0.84	0.80	0.04 [−0.33 to 0.42]	0.82
Sleep latency	1.66	1.48	0.18 [−0.44 to 0.81]	0.55
Daytime dysfunctions	0.95	0.95	0.00 [−0.50 to 0.49]	0.99
Sleep disturbances	1.58	1.56	0.02 [−0.31 to 0.35]	0.90
Use of sleep medication				
*QOL*				
MCS	47.41	51.30	−3.88 [−8.63 to 0.87]	0.11
PCS	44.39	41.94	2.45 [−1.69 to 6.58]	0.24

PSQI, Pittsburgh Sleep Quality Index; QOL, quality of life; MCS, mental component summary; PCS, physical component summary. *Note*.  ^*∗*^Results for subscale “use of sleep medication” were not included due to a lack of variability in the data.

**Table 3:** Individual scales of TAQ at four weeks.

Individual scales of TAQ	Intervention (*n* = 21)	Control (*n* = 19)	*p* values
Question 1: acceptability	7 [6, 7]	6 [6, 7]	0.41
Question 2: efficacy	6 [5, 7]	6 [4, 7]	0.90
Question 3: side effects	1 [1, 1]	1 [1, 1]	0.43
Question 4: trust rank of the therapist	7 [7, 7]	7 [7, 7]	0.23

TAQ: treatment acceptability questionnaire.

Note: values are given as median [interquartile range].

For Question  1, 1 represents “very unacceptable” and 7 represents “very acceptable”; for Question  2, 1 represents “very ineffective” and 7 represents “very effective”; for Question  3, 1 represents “very unlikely” and 7 represents “very likely”; for Question  4, 1 represents “very untrustworthy” and 7 represents “very trustworthy.”

**Table 4:** Listing of SAEs.

SAEs	Treatment group	Body system for SAE
Fluid overload	Intervention	Cardiovascular
Eye haemorrhage	Intervention	Cardiovascular
Necrotising Fasciitis	Control	Musculoskeletal
Physical trauma	Control	Musculoskeletal
Arteriovenous graft failure	Control	Musculoskeletal
Chest muscle pain	Control	Other

SAE: serious adverse event.
