# Supplementary material for: The SIESTA Trial: A Randomized Study Investigating the Efficacy, Safety, and Tolerability of Acupressure versus Sham Therapy for Improving Sleep Quality in Patients with End-Stage Kidney Disease on Hemodialysis
Source: Evid Based Complement Alternat Med. 2017 Feb 21;2017:7570352. doi: 10.1155/2017/7570352 (PMC5339536; doi:10.1155/2017/7570352)
Supplement: Supplementary file 1 — The supplementary material shows all the questionnaires that were employed in the SIESTA study. Supplementary appendix 1 shows the sleep diary designed to reflect sleep patterns of patients on haemodialysis, including elements that may disturb their nocturnal sleep (i.e. pain and caffeine intake) in Supplementary Table S1. The Pittsburg Sleep Quality Index (PSQI) instrument is found in supplementary appendix 2; and the Short form-8 (SF-8) Health Survey Scoring Demonstration in supplementary appendix 3. Indications of acupoints stimulated in the study are summarized in a figures clarifying the locations, in supplementary appendix 4, and the Treatment Acceptability Questionnaire (TAQ) is found in supplementary appendix 5. The results of the treatment group differences in the main and secondary outcomes when additionally adjusted for another two confounding factors, duration of dialysis and hours per dialysis session, are in Supplementary Table S2. [file 7570352.f1.pdf]

Supplementary Appendix 1. Sleep diary

Start date: __/__/2015 Complete in Morning							
Day of week:	Day 1	Day 2	Day 3	Day 4	Day 5	Day 6	Day 7
I went to bed last night at	PM/ AM	PM/ AM	PM/ AM	PM/ AM	PM/ AM	PM/ AM	PM/ AM
I got out of bed this morning at	PM/ AM	PM/ AM	PM/ AM	PM/ AM	PM/ AM	PM/ AM	PM/ AM
Last night I fell asleep:							
Easily	☐	☐	☐	☐	☐	☐	☐
After sometime	☐	☐	☐	☐	☐	☐	☐
With difficulty	☐	☐	☐	☐	☐	☐	☐
I woke up during the night:							
# of times							
last night I slept a total of:	Hours	Hours	Hours	Hours	Hours	Hours	Hours
My sleeps were disturbed by:							
Pain	☐	☐	☐	☐	☐	☐	☐
Itch	☐	☐	☐	☐	☐	☐	☐
Short of breath	☐	☐	☐	☐	☐	☐	☐
Thirst/dry mouth	☐	☐	☐	☐	☐	☐	☐
Waking up to pass urine	☐	☐	☐	☐	☐	☐	☐
Other (please indicate reason)							
When I woke up for the day, I felt							

**Refreshed**	☐	☐	☐	☐	☐	☐	☐
**Somewhat refreshed**	☐	☐	☐	☐	☐	☐	☐
**Fatigued**	☐	☐	☐	☐	☐	☐	☐

Complete at the End of Day

**Day of week:**	Day 1	Day 2	Day 3	Day 4	Day 5	Day 6	Day 7
**I exercised at least 20 minutes in the:**							
**Morning**	☐	☐	☐	☐	☐	☐	☐
**Afternoon**	☐	☐	☐	☐	☐	☐	☐
**Evening**	☐	☐	☐	☐	☐	☐	☐
**N/A**	☐	☐	☐	☐	☐	☐	☐
**During the day, how likely was I to doze off while performing daily activities:**							
**No chance**	☐	☐	☐	☐	☐	☐	☐
**Low chance**	☐	☐	☐	☐	☐	☐	☐
**High chance**	☐	☐	☐	☐	☐	☐	☐
**N/A**	☐	☐	☐	☐	☐	☐	☐
**During the day, my mood was:**							
**Very Pleasant**	☐	☐	☐	☐	☐	☐	☐
**Pleasant**	☐	☐	☐	☐	☐	☐	☐
**Unpleasant**	☐	☐	☐	☐	☐	☐	☐

**Very unpleasant**	☐	☐	☐	☐	☐	☐	☐
**Took a nap?**	yes<br>☐	yes<br>☐	yes<br>☐	yes<br>☐	yes<br>☐	yes<br>☐	yes<br>☐
	no<br>☐	no<br>☐	no<br>☐	no<br>☐	no<br>☐	no<br>☐	no<br>☐
**Compete at the End of Day**							
**Approximately 2-3 hours before going to bed, I consumed:**							
**Cigarettes**	☐	☐	☐	☐	☐	☐	☐
**Alcohol**	☐	☐	☐	☐	☐	☐	☐
**Caffeine**	cocoa<br>☐	cocoa<br>☐	cocoa<br>☐	cocoa<br>☐	cocoa<br>☐	cocoa<br>☐	cocoa<br>☐
	cola<br>☐	cola<br>☐	cola<br>☐	cola<br>☐	cola<br>☐	cola<br>☐	cola<br>☐
	tea<br>☐	tea<br>☐	tea<br>☐	tea<br>☐	tea<br>☐	tea<br>☐	tea<br>☐
	coffee<br>☐	coffee<br>☐	coffee<br>☐	coffee<br>☐	coffee<br>☐	coffee<br>☐	coffee<br>☐
**In the hour before going to sleep, my bedtime routine includes:**							
**Use of electronics (e.g. phone, laptop)**	yes<br>☐	yes<br>☐	yes<br>☐	yes<br>☐	yes<br>☐	yes<br>☐	yes<br>☐
	no<br>☐	no<br>☐	no<br>☐	no<br>☐	no<br>☐	no<br>☐	no<br>☐
**Relaxation exercises**	☐	☐	☐	☐	☐	☐	☐
**Took sleep medications?**	yes	yes	yes	yes	yes	yes	yes

	☐	☐	☐	☐	☐	☐	☐
	**no** ☐	**no** ☐	**no** ☐	**no** ☐	**no** ☐	**no** ☐	**no** ☐

Supplementary Appendix 2. PSQI instrument

Subject's Initials_______________ID#_________________Date______________Time_____ AM PM

PITTSBURGH SLEEP QUALITY INDEX

INSTRUCTIONS:
The following questions relate to your usual sleep habits during the past month only. Your answers should indicate the most accurate reply for the majority of days and nights in the past month. Please answer all questions.

1. During the past month, what time have you usually gone to bed at night?

   BED TIME ___________

2. During the past month, how long (in minutes) has it usually taken you to fall asleep each night?

   NUMBER OF MINUTES ___________

3. During the past month, what time have you usually gotten up in the morning?

   GETTING UP TIME ___________

4. During the past month, how many hours of actual sleep did you get at night? (This may be different than the number of hours you spent in bed.)

   HOURS OF SLEEP PER NIGHT ___________

For each of the remaining questions, check the one best response. Please answer all questions.

5. During the past month, how often have you had trouble sleeping because you . . .

a) Cannot get to sleep within 30 minutes

Not during the past month_____ Less than once a week_____ Once or twice a week_____ Three or more times a week_____

b) Wake up in the middle of the night or early morning

Not during the past month_____ Less than once a week_____ Once or twice a week_____ Three or more times a week_____

c) Have to get up to use the bathroom

Not during the past month_____ Less than once a week_____ Once or twice a week_____ Three or more times a week_____

d) Cannot breathe comfortably

Not during the past month_____	Less than once a week_____	Once or twice a week_____	Three or more times a week_____

e) Cough or snore loudly

Not during the past month_____	Less than once a week_____	Once or twice a week_____	Three or more times a week_____

f) Feel too cold

Not during the past month_____	Less than once a week_____	Once or twice a week_____	Three or more times a week_____

g) Feel too hot

Not during the past month_____	Less than once a week_____	Once or twice a week_____	Three or more times a week_____

h) Had bad dreams

Not during the past month_____	Less than once a week_____	Once or twice a week_____	Three or more times a week_____

i) Have pain

Not during the past month_____	Less than once a week_____	Once or twice a week_____	Three or more times a week_____

j) Other reason(s), please describe____

____

How often during the past month have you had trouble sleeping because of this?

Not during the past month_____	Less than once a week_____	Once or twice a week_____	Three or more times a week_____

6. During the past month, how would you rate your sleep quality overall?

Very good ____________

Fairly good ____________

Fairly bad ____________

Very bad ____________

7. During the past month, how often have you taken medicine to help you sleep (prescrit "over the counter")?

Not during the past month_____ Less than once a week_____ Once or twice a week_____ Three or more times a week_____

8. During the past month, how often have you had trouble staying awake while driving, meals, or engaging in social activity?

Not during the past month_____ Less than once a week_____ Once or twice a week_____ Three or more times a week_____

9. During the past month, how much of a problem has it been for you to keep up e enthusiasm to get things done?

No problem at all ___________

Only a very slight problem ___________

Somewhat of a problem ___________

A very big problem ___________

10. Do you have a bed partner or room mate?

No bed partner or room mate ___________

Partner/room mate in other room ___________

Partner in same room, but not same bed ___________

Partner in same bed ___________

If you have a room mate or bed partner, ask him/her how often in the past month you have had . . .

a) Loud snoring

Not during the past month_____ Less than once a week_____ Once or twice a week_____ Three or more times a week_____

b) Long pauses between breaths while asleep

Not during the past month_____ Less than once a week_____ Once or twice a week_____ Three or more times a week_____

c) Legs twitching or jerking while you sleep

Not during the past month_____ Less than once a week_____ Once or twice a week_____ Three or more times a week_____

d) Episodes of disorientation or confusion during sleep

Not during the past month_____	Less than once a week_____	Once or twice a week_____	Three or more times a week_____

e) Other restlessness while you sleep; please describe________________________________

____

Not during the past month_____	Less than once a week_____	Once or twice a week_____	Three or more times a week_____

© 1989, University of Pittsburgh. All rights reserved. Developed by Buysse,D.J., Reynolds,C.F., Monk,T.H., Berman,S.R., and Kupfer,D.J. of the University of Pittsburgh using National Institute of Mental Health Funding.

Buysse DJ, Reynolds CF, Monk TH, Berman SR, Kupfer DJ: Psychiatry Research, 28:193-213, 1989.

Supplementary Appendix 3. SF-8^{TM} Health Survey Scoring Demonstration

This survey asks for your view about your health. This information will help you keep track of how you feel and how well you are able to do your usual activities. Thank you for completing this survey!

Answer every question by selecting the answer as indicated. If you are unsure about how to answer a question, please give the best answer you can.

For each of the following questions, please mark an[x] in the one box that best describes your answer.

1. Overall, how would you rate your health during the past 4 weeks?

   Excellent Very good Good Fair Poor Very poor

2. During the past 4 weeks, how much did physical health problems limit your usual physical activities (such as walking or climbing stairs)?

   Not at all Very little Somewhat Quite a lot Could not do physical activities

3. During the past 4 weeks, how much difficulty did you have doing your daily work, both at home and away from home, because of your physical health?

   None at all A little bit Some Quite a lot Could not do daily work

4. How much bodily pain have you had during the past 4 weeks?

   None Very mild Mild Moderate Severe Very Severe

5. During the past 4 weeks, how much energy did you have?

   Very much Quite a lot Some A little None

6. During the past 4 weeks, how much did your physical health or emotional problems limit your usual social activities with family or friends?

   Not at all Very little Somewhat Quite a lot Could not do social activities

7. During the past 4 weeks, how much have you been bothered by emotional problems (such as feeling anxious, depressed or irritable)?

   Not at all Slightly Moderately Quite a lot Extremely

8. During the past 4 weeks, how much did personal or emotional problems keep you from doing your usual work, school or other daily activities?

Not at all   Very little   Somewhat   Quite a lot   Could not do daily activities

Thank you for completing these questions!

Supplementary Appendix 4. Locations and indications of the acupoints, non-specific acupoint and sham acupoint

Name	Location	Indications	Pictures
**HT7**<br><br>**(Real - acupoints)**	At the ulnar end of the transverse crease of the wrist, in the depression on the radial side of the tendon of m. flexor carpi ulnaris	Cardiac pain, irritability, palpitation, hysteria, amnesia, insomnia, epilepsy, dementia, pain in the hypochondriac region, feverish sensation in the palm, yellowish sclera	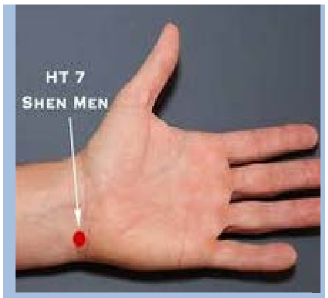
**K11**<br><br>**(Real - acupoints)**	On the sole of the foot, in the depression when the foot is in plantar flexion, approximately at the junction of the anterior one-third and posterior two-thirds of the line connecting the base of the second and third toes and the heel	Headache, blurring of vision, dizziness, sore throat, dryness of tongue, loss of voice, dysuria, infantile convulsion, feverish sensation in the sole of the foot, loss of consciousness	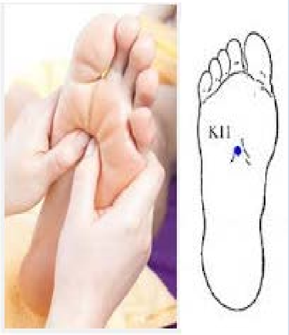
**EX-EU3**<br><br>**(Non-specific acupoint)**	Pronate one's palm; on the transverse striae of dorsal carpus, between tendons of extensor muscle and short radial extensor muscle of wrist	Stuffy sensation in the chest, gastric pain, and with blood sputum in certain cases	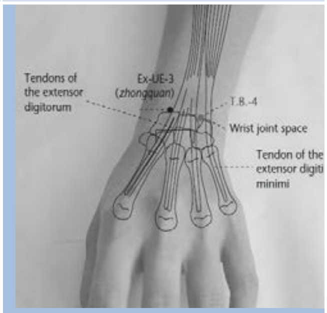
**S**<br><br>**(Sham - acupoints)**	1 cm right to K11	NA	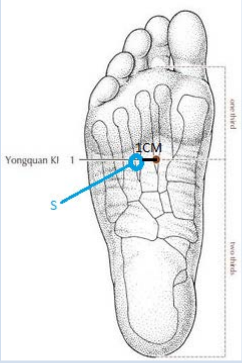

Zhu, B.M.D., H. Wang, and C. Ebooks, *Meridians and acupoints*. 2010, London; Philadelphia: SingingDragon.

Supplementary Appendix 5. Treatment Acceptability Questionnaire (TAQ)

Please answer these questions that deal with your reactions to the acupressure therapy. Circle the number that best describes your reactions.

1. Overall, how acceptable do you find the treatment was?

   VERY UNACCEPTABLE 1 2 3 4 5 6 7 VERY ACCEPTABLE

2. How effective do you think this treatment is to be?

   VERY INEFFECTIVE 1 2 3 4 5 6 7 VERY EFFECTIVE

3. How likely do you think this treatment may have negative side effects?

   VERY UNLIKELY 1 2 3 4 5 6 7 VERY LIKELY

4. How trustworthy do you think the therapist was?

   VERY UNTRUSTWORTHY 1 2 3 4 5 6 7 VERY TRUSTWORTHY

Supplementary Table S1. Sleep Diary – Baseline and End of Study

	Baseline			End of study		
	Intervention (n=20)	control (n=20)	P Values	Intervention (n=21)	Control (n=19)	P Values
Duration of sleep (hours/night)[b]	6.3 ±1.5	6.3 ± 1.4	0.85	6.6 ± 2.1	7.0 ± 1.3	0.52
Number of times waking up during sleep [b]	1.6 ± 1.4	1.4 ± 1.3	0.66	1.4 ± 1.2	1.5 ± 1.3	0.67
**Difficulty degree of falling asleep during the week [b]**						
Number of days/week with easiness in falling asleep	4 [0,7]	3 [0,5.5]	0.83	3 [1,6]	4 [2,7]	0.50
Number of days/week with difficulty in falling asleep	0 [0,2.5]	0.5 [0,2]	0.77	0 [0,1]	1 [0,2]	0.30
**Spiritual status right after waking up during the week [b]**						
Number of days/week waking up feeling refreshed	2 [0,6.5]	0 [0,3]	0.20	2.5±2.7	2.5±2.7	1.0
Number of days/week waking up feeling somewhat refreshed	2.1± 2.4	2.7 ± 2.4	0.40	2.9 ± 2.7	2.4 ± 2.3	0.58
Number of days/week waking up feeling fatigued	2.0 ± 2.8	2.45 ± 2.5	0.55	1 [0,2]	1 [0,2]	0.99
**Chance of dozing off during the week [b]**						
Number of days/week with no chance to doze off while performing daily activities	4.5 [1,7]	0.5 [0,4]	0.03	5 [0,7]	3 [0,7]	0.99
Number of days/week with low chance to doze off while performing daily activities	0 [0,3]	1.5 [0,6.5]	0.19	0 [0,1]	0 [0,2]	0.93
Number of days/week with high chance to doze off while performing daily activities	0 [0,1]	0 [0,2.5]	0.50	0 [0,1]	0 [0,3]	0.39
**Mood during the week [b]**						
Number of days/week being very pleasant	1 [0,5]	0 [0,1]	0.08	0 [0,2]	2 [0,5]	0.10

Number of days/week being pleasant	1.5 [0,6.5]	5.5 [4,7]	0.05	4 [2,7]	4 [1,7]	0.56
Number of days/week being unpleasant	0 [0,1]	0 [0,1]	0.50	0 [0,1]	0 [0,1]	0.95
Number of days/week being very unpleasant	0 [0,0]	0 [0,0]	0.08	0 [0,0]	0 [0,0]	0.17
**Exercise status during the week** [b]						
Taking exercises over 3 hours/week	4 (20%)	7 (35%)	0.29	4 (19.1%)	3 (15.8%)	0.79
**Habits of taking a nap during the week** [b]						
Never taking a nap during the week	5 (25%)	7 (35%)	0.49	5 (23.8%)	8 (42.1%)	0.22
Taking a nap≥4 days/week	8 (40%)	8 (40%)	1	10 (47.6%)	4 (21.1%)	0.08
**Habits before going to sleep during the week**						
**Cigarettes taking status** [b]						
Never smoking 2-3 hours before sleep during the week	17 (85%)	17 (85%)	1	17 (81.0%)	18 (94.7%)	0.19
Smoking 2-3 hours before sleep ≥4 days/week	3 (15%)	3(15%)	1	3 (14.3%)	1 (5.3%)	0.34
**Alcohol drinking status** [b]						
Never drinking alcohol 2-3 hours before sleep during the week	20 (100%)	18 (90%)	0.15	21 (100%)	19 (100%)	1
Drinking alcohol 2-3 hours before sleep ≥4 days/week	0 (0%)	1 (5%)	0.31	0 (0%)	0 (0%)	1.000
**Caffeine taking status** [b]						
Never taking beverage containing caffeine 2-3 hours before sleep during the week	7 (35%)	10 (50%)	0.34	13 (61.9%)	13 (68.4%)	0.67
Taking beverage containing caffeine 2-3 hours before sleep ≥4 days/week	9 (45%)	7 (35%)	0.52	7 (33.3%)	5 (26.3%)	0.63
**Electronics (e.g. smartphone, laptop, TV, radio) using status** [b]						
Never using electronic devices one hour before sleep during the week	9 (45%)	13 (65%)	0.20	12 (57.1%)	10 (52.6%)	0.78

Using electronic devices one hour before sleep ≥4 days/week	9 (45%)	6 (30%)	0.33	5 (23.8%)	9 (47.4%)	0.12
**Relaxation exercise taking status** [b]						
Never taking relaxation exercises before sleep	18 (90%)	17 (85%)	0.63	20 (95.2%)	17 (89.5%)	0.49
Taking relaxation exercise before sleep ≥4 days/week	2 (10%)	2 (10%)	1.00	1 (4.8%)	2 (10.5%)	0.49
**Sleep aided by medications during the week** [b]						
Number of days/week taking medicine to help sleep	0 [0,0.5]	0 [0,0]	0.09	0 [0,0]	0 [0,0]	0.20
**Symptoms disturbing sleep during the week** [b] -Number of days/week sleep disturbed by:						
Pain	0 [0,3.5]	0 [0,3]	0.58	0 [0,2]	0 [0,5]	0.45
Itch	0 [0,0]	0 [0,0]	0.46	0 [0,0]	0 [0,0]	0.14
Short of breath	0 [0,0]	0 [0,0]	1	0 [0,0]	0 [0,0]	0.23
Thirst	0 [0,0]	0.5 [0,7]	0.09	0 [0,0]	0 [0,1]	0.84
Waking up to pass urine	0 [0,1]	0.5 [0,7]	0.16	0 [0,1]	0 [0,4]	0.29
Others						
Restless legs syndrome	0 [0,0]	0 [0,0]	1	0 [0,0]	0 [0,0]	0.34
Cramps	0 [0,0]	0 [0,0]	0.16	0 [0,0]	0 [0,0]	0.34
Vomiting/reflux	0 [0,0]	0 [0,0]	1	0 [0,0]	0 [0,0]	0.13

Note:

[b]One missing value in the intervention group at baseline.

Values for categorical variables are given as number (percentage); values for continuous variables are given as mean ±SD if normally distributed, or if non-normally as median [interquartile range]. Data was submitted to two-tail student t-test if assumption of normality distribution was met, otherwise, Mann-Whitney test was used to compare the difference between groups.

Supplementary Table S2. Primary (PSQI) and secondary (QOL) outcomes at four weeks by treatment group (adjusted for baseline values, duration of dialysis and hours per dialysis session.

**Outcome**	**Intervention (N = 21)**	**Control (N = 19)**	**Difference (Intervention - Control) [95% CI]**	**P-value**
**PSQI global scores**	7.31	7.76	-0.45 [-2.15 to 1.26]	0.60
***PSQI subscales**				
Sleep duration	1.05	1.10	-0.05 [-0.49 to -0.40]	0.84
Habitual sleep efficiency	1.07	1.29	-0.22 [-0.85 to 0.41]	0.48
Subjective sleep quality	0.80	0.86	-0.06 [-0.48 to 0.37]	0.79
Sleep latency	1.51	1.65	-0.14 [-0.82 to 0.55]	0.69
Daytime dysfunctions	1.00	0.90	0.10 [-0.48 to 0.67]	0.74
Sleep disturbances	1.50	1.66	-0.16 [-0.53 to 0.21]	0.38
**QOL**				
MCS	46.87	51.90	-5.03 [-10.43 to 0.38]	0.07
PCS	44.79	41.50	3.29 [-1.48 to 8.06]	0.17

Abbreviations: PSQI, Pittsburgh Sleep Quality Index; QOL, quality of life; MCS, mental component summary; PCS, physical component summary.

*Results for subscale “use of sleep medication” were not included due to a lack of variability in the data.
